# Aggregate-Reactivation Activity of the Molecular Chaperone ClpB from *Ehrlichia chaffeensis*

**DOI:** 10.1371/journal.pone.0062454

**Published:** 2013-05-07

**Authors:** Ting Zhang, Sabina Kedzierska-Mieszkowska, Huitao Liu, Chuanmin Cheng, Roman R. Ganta, Michal Zolkiewski

**Affiliations:** 1 Department of Biochemistry and Molecular Biophysics, Kansas State University, Manhattan, Kansas, United States of America; 2 Department of Biochemistry, University of Gdansk, Gdansk, Poland; 3 Department of Diagnostic Medicine/Pathobiology, Kansas State University, Manhattan, Kansas, United States of America; Washington State University, United States of America

## Abstract

Rickettsiale diseases, including human monocytic ehrlichiosis caused by *Ehrlichia chaffeensis*, are the second leading cause of the tick-borne infections in the USA and a growing health concern. Little is known about how *E. chaffeensis* survives the host-induced stress in vertebrate and tick hosts. A molecular chaperone ClpB from several microorganisms has been reported to reactivate aggregated proteins in cooperation with the co-chaperones DnaK/DnaJ/GrpE (KJE). In this study, we performed the first biochemical characterization of ClpB from *E. chaffeensis*. The transcript of *E. chaffeensis* ClpB (EhClpB) is strongly upregulated after infection of cultured macrophages and its level remains high during the *Ehrlichia* replicative stage. EhClpB forms ATP-dependent oligomers and catalyzes the ATP hydrolysis, similar to *E. coli* ClpB (EcClpB), but its ATPase activity is insensitive to the EcClpB activators, casein and poly-lysine. EhClpB in the presence of *E. coli* KJE efficiently reactivates the aggregated glucose-6-phosphate dehydrogenase (G6PDH) and firefly luciferase. Unlike EcClpB, which requires the co-chaperones for aggregate reactivation, EhClpB reactivates G6PDH even in the absence of KJE. Moreover, EhClpB is functionally distinct from EcClpB as evidenced by its failure to rescue a temperature-sensitive phenotype of the *clpB-*null *E. coli*. The *clpB* expression pattern during the *E. chaffeensis* infection progression correlates with the pathogen’s replicating stage inside host cells and suggests an essential role of the disaggregase activity of ClpB in the pathogen’s response to the host-induced stress. This study sets the stage for assessing the importance of the chaperone activity of ClpB for *E. chaffeensis* growth within the mammalian and tick hosts.

## Introduction

Rikettsiales pathogens of the genera *Ehrlichia* and *Anaplasma* have been identified in recent years as a growing health concern and are the second leading cause of the tick-borne illnesses in the USA after *Borrelia burgdorferi*, which causes Lyme disease. *Ehrlichia chaffeensis* is an intracellular pathogen transmitted through an infected tick, *Amblyomma americanum,* to humans and several other vertebrate hosts [1]–[8]. The pathogen is responsible for causing human monocytic ehrlichiosis (HME) [6], [7], [9]. The disease is characterized by an acute onset of febrile illness that can progress to a fatal outcome, particularly in immune compromised individuals [2].

*E. chaffeensis* has an unusual developmental cycle that requires growth and replication within phagosomes of eukaryotic cells of vertebrate and tick hosts [10]. During its developmental cycle, there is a conversion between two distinct morphological forms, the elementary bodies (EBs) and the reticulate bodies (RBs) [11], [12]. EBs are the infectious form of *E. chaffeensis*; they convert into metabolically active RBs upon entry into a host cell. RBs are larger than EBs and divide by binary fission [11], [13], [14]. Later in the developmental cycle, RBs convert back to EBs, which are released from infected cells [11], [13], [14]. The transformation of the *E. chaffeensis* RBs to EBs has been observed in both vertebrate and tick hosts [14], [15]. It is not clear how the pathogen overcomes the host-induced stress in support of its invasion and replication in host phagosomes and the subsequent release and reinfection of naïve host cells.

The role of stress and stress-response in host-pathogen interactions is emerging as a novel direction in studies on the mechanisms of infection [16], [17]. Importantly, proteins that mediate responses to stress can become targets for novel antimicrobial therapies [18]. However, a successful antibiotic targeting of the pathogenic stress-response machinery has not been accomplished yet, mostly due to strong sequence conservation among stress-induced proteins from different organisms and their essential role in protein homeostasis in all forms of life.

ClpB is an ATP-dependent molecular chaperone from the Hsp100 family that disaggregates and reactivates aggregated proteins accumulating under conditions of stress [19]. For all Hsp100 chaperones investigated so far, the unique disaggregase activity requires cooperation with the members of Hsp70 and Hsp40 families (bacterial DnaK/DnaJ) [20]–[23]. Hsp100 chaperones belong to the AAA+ super-family of ATPases associated with different cellular activities, a group of energy-dependent conformation-remodeling factors [24], [25]. ClpB is produced in prokaryotes, fungi (where it is known as Hsp104), and plants, but, unlike other chaperones, it is not found in animals and humans. ClpB is dispensable under normal growth conditions, but becomes essential for survival under severe stress [26], [27]. Importantly, ClpB is required for invasiveness and/or in-host survival of a number of bacterial and protozoan pathogens including *Porphyromonas gingivalis* [28], *Mycoplasma pneumoniae* [29], *Francisella tularensis* [30], *Enterococcus faecalis* [31], *Mycobacterium tuberculosis* [32], *Leptospira interrogans* [33], and *Leischmania donovani* [34]. The apparent lack of metazoan ClpB orthologs makes this chaperone an attractive target for developing novel antimicrobial therapies. The mechanism of ClpB from non-pathogenic organisms has been extensively studied during the last decade (for reviews see [19], [35], [36]), but no information about the activity and function of ClpB in pathogens is available.

We report the first biochemical characterization of ClpB from a pathogenic microorganism, *E. chaffeensis* (EhClpB, NCBI accession number YP_507187). We discovered that the *clpB* expression is high during the pathogen’s replication stage of the infection cycle. A recombinant EhClpB shows the aggregate-reactivation activity *in vitro*. Importantly, EhClpB displays a distinct linkage between the nucleotide and substrate binding that has not been observed in other Hsp100 proteins and translates into a unique capability of EhClpB to mediate protein disaggregation independently from the Hsp70/40 co-chaperones. This work provides a critical first step for assessing the importance of the disaggregase activity of ClpB for *E. chaffeensis* growth within the mammalian and tick hosts.

## Materials and Methods

### Proteins

The pET28a vector (Novagen) encoding *E. chaffeensis clpB* was used to produce the recombinant EhClpB. The entire protein coding sequence of *E. chaffeensis clpB* was amplified using Pfu DNA polymerase (Promega) with the gene-specific PCR primers (ClpB_forward primer: 5′CACCATATGgatctcaatcaatttactgatatg, with the NdeI site underlined, and ClpB_reverse: 5′CGACTCGAGctataacttattaataattaaatcgtcattc, with the XhoI site underlined) using the pathogen genomic DNA as the template. The PCR product was digested with NdeI and XhoI, purified to remove the digested overhangs, and ligated with the similarly digested pET28a plasmid to produce pET28a-EhClpB. Recombinant EhClpB from this plasmid construct was produced in the *E. coli* strain Rosetta™ 2(DE3) (EMD Millipore). The *E. coli* cells were grown at 37°C until the optical density at 600 nm reached ∼0.5 and then induced with 0.4 mM IPTG for 2 h. The cells were then collected, disrupted by sonication and centrifuged to collect the soluble extract. The soluble fraction was treated with 4 mg/(g cells) polyethyleneimine (PEI). After centrifugation (20,000 g, 1 h), the supernatant was applied to a Ni-NTA column (Invitrogen) and the bound protein was eluted with 250 mM imidazole. Fractions containing EhClpB were identified with SDS-PAGE/Coomassie stain, combined, and further purified by gel filtration on Superdex®200 (GE LifeSciences). The N-terminal His-tag was removed with Thrombin Cleavage Capture Kit (Novagen). The identity of the purified EhClpB was confirmed with an MS analysis of tryptic peptides, performed at the Biotechnology/Proteomics Core Facility at KSU. The post-cleavage N-terminal sequence of the recombinant EhClpB contains three additional amino acids GlySerHis.

*E. coli* chaperones (EcClpB, DnaK, DnaJ, GrpE) were produced or obtained as previously described [37]. Glucose-6-phosphate dehydrogenase (G6PDH) from *Leuconostoc mesenteroides* was produced as described before [38]. Firefly luciferase was obtained from Promega and κ-casein from Sigma. Protein concentration was determined spectrophotometrically and reported in monomer units.

### *In Vitro* Cultivation of *E. chaffeensis*

The canine macrophage cell line, DH82, has been continuously cultivated under *in vitro* conditions. DH82 is a macrophage-monocyte cell line from a dog with malignant histiocytosis [39] and is commonly used for *in vitro* cultivation of *E. chaffeensis* [40]. The cell line is commercially available at ATCC (Catalog # CRL-10389). The culture medium consisted of 500 ml minimum essential medium with Earle’s salt, 6 ml 200 mM L-glutamine, and 35 ml heat-inactivated fetal bovine serum. Cells were incubated at 37°C with 5% CO_2_. Nine confluent T25 flasks containing healthy DH82 cells were each inoculated with 100 µl of macrophage culture-derived Arkansas isolate of *E. chaffeensis* dense core infectious form. Cultures from one flask each were recovered at each of the following time points; 0, 6, 12, 24, 36, 48, 60, 72, and 84 hrs post-infection by centrifugation at 15,000 g for 15 min. The resulting pellets were dissolved in 0.5 ml of TRI-Reagent and stored at −80°C or used immediately for RNA isolation.

### RNA Isolation

Total RNA was isolated using the TRI-Reagent method (Sigma Aldrich) according to the manufacturer’s instructions. RNeasy Mini Kit (Qiagen) was used to remove a residual contaminating genomic DNA. The final purified RNA from each flask was resuspended in 50 µl of nuclease-free water.

### Complementary DNA (cDNA) Synthesis

cDNA was synthesized from the RNA samples by using random hexamers and reverse transcriptase as described in the SuperScript III First-Strand Synthesis System for RT-PCR kit (Invitrogen). The concentrations of cDNA from each time point of the recovered sample were estimated by the nano-drop method, their abundance was estimated by performing TaqMan-based real time PCR assay targeted to the 16 S rDNA. Subsequently, the concentrations of cDNAs were adjusted to represent equal numbers of 16 S rDNA molecules per µl of solution.

### Determination of the *E. chaffeensis* ClpB, ClpA and DnaK Gene Expression by Semi-Quantitative RT-PCR

Gene-specific primers targeting ClpB cDNA (RRG839, 5′ tta cct gtt gta agt gga agt gg, and RRG840, 5′ ctt aca cga ctt gct tca tc), ClpA cDNA (RRG841, 5′ gctagtttacacaaggcactgtc, and RRG842, 5′ cacgatagcgtgttccagc), or DnaK cDNA (RRG795, 5′ tacagctgctgctttggcgtatg and RRG796, 5′ cacccttatgaggttctctacc) were used in the PCR analysis using cDNAs as the templates. The PCRs were performed using 2 µl each of the concentration adjusted cDNA solutions added to 23 µl of master mix containing 1x platinum qPCR super mix, 5 pmol of each of the forward and reverse primers as per the manufacturer’s instruction (Invitrogen). Amplifications were performed at varying PCR cycles ranging from 20–40 cycles to estimate the variations in the expression levels. The amplicons were resolved on a 1.0% agarose gel and captured with Kodak Gel Logic 200 imaging system.

### Analytical Ultracentrifugation

Beckman XL-I analytical ultracentrifuge was used in sedimentation velocity experiments with two-channel analytical cells. Ultracentrifugation was performed at 48,000 rpm and 20°C for the 0.3-mg/ml protein samples in 50 mM Hepes/KOH pH 7.5, 0.2 M KCl, 20 mM MgCl_2_, 1 mM EDTA, 2 mM β-mercaptoethanol without and with 2 mM ATPγS (adenosine-5'-(γ-thio)-triphosphate). The data were analyzed using the time-derivative method [41], [42] and the software distributed with the instrument.

### ClpB ATPase Activity

EhClpB and EcClpB were incubated in assay buffer (100 mM Tris/HCl pH 8.0, 1 mM DTT, 1 mM EDTA, 10 mM MgCl_2_, and 5 mM ATP) at 37°C for 15 min without or with 0.1 mg/ml κ -casein or 0.04 mg/ml poly-lysine, or 2 µM aggregated G6PDH. The concentration of ClpB was 0.05 mg/ml for the basal activity and in the presence of κ -casein and G6PDH or 0.005 mg/ml in the presence of poly-lysine. The concentration of phosphate generated by ClpB was measured as described before [21].

### Aggregate Reactivation Assays

To produce aggregates of G6PDH, the protein stock (320 µM) was diluted 2-fold with the unfolding buffer (10 M urea, 16% glycerol and 40 mM DTT) and incubated at 47°C for 5 min. Then, the unfolded G6PDH was diluted 10-fold with the refolding buffer 1 (50 mM Tris/HCl pH 7.5, 20 mM Mg(OAc)_2_, 30 mM KCl, 1 mM EDTA, and 1 mM β-mercaptoethanol) and incubated at 47°C for 15 min and then on ice for 2 min to arrest the aggregation. Aggregated G6PDH (16 µM) was diluted 10-fold with refolding buffer 1 containing 1.5 µM ClpB, 1 µM DnaK, 1 µM DnaJ, 0.5 µM GrpE and 6 mM ATP. The sample was incubated at 30°C and aliquots were withdrawn to test the recovery of the G6PDH enzymatic activity. Aggregates diluted with refolding buffer without the chaperones were used as control. To measure the G6PDH activity, aliquots from the refolding reaction were incubated in 50 mM Tris/HCl pH 7.8, 5 mM MgCl_2_, 1.5 mM G6P and 1 mM NADP^+^ for 10 min followed by the measurement of absorption at 340 nm. To produce aggregates of firefly luciferase, 220 µM luciferase stock was diluted 300-fold with PBS containing 1 mg/ml BSA and then incubated at 45°C for 12 min. Aggregated luciferase (1 µM) was diluted 20-fold with refolding buffer 2 (30 mM Hepes, pH 7.65, 120 mM KCl, 10 mM MgCl_2_, 6 mM ATP, 1 mM EDTA, 10 mM DTT, 0.1 mg/ml BSA) containing 1.5 µM ClpB, 1 µM DnaK, 1 µM DnaJ, and 0.5 µM GrpE. The mixture was incubated at room temperature and aliquots were withdrawn to test the recovery of the luciferase activity using the luminescence assay system (Promega).

### ClpB-Aggregate Interaction Assay

Aggregated G6PDH (16 µM) was diluted 10-fold with the refolding buffer 1 containing 1.5 µM ClpB and 6 mM nucleotide (ADP, ATP, AMPPNP [adenylyl-imidodiphosphate], or ATPγS). The sample was incubated at 30°C with 600 rpm shaking for 20 min and then was applied to the filter device (Millipore Ultrafree-MC Centrifugal Filter Unit with the membrane pore size 0.1 µm). After 5 min incubation at room temperature, the filter device was centrifuged at 13,000 rpm for 4 min. The filter device was then washed with the refolding buffer 1 containing the appropriate nucleotide at 30°C for 5 min and then re-centrifuged. Next, 1x SDS-loading buffer was added to the filter device and the filter device was incubated at 50°C for 5 min with shaking. Then, the filter device was centrifuged to obtain the eluate fractions, which were applied to SDS-PAGE. Aggregated luciferase (7 µM) was diluted 20-fold with refolding buffer 2 (30 mM Hepes, pH 7.65, 120 mM KCl, 10 mM MgCl_2_, 1 mM EDTA, 10 mM DTT) containing 1.5 µM ClpB with 5 mM nucleotide and processed as described above.

### Heat-Shock Survival Assay

The *E. chaffeensis clpB* DNA sequence was subcloned into a low-copy pGB2 plasmid downstream from the native *E. coli* ClpB heat-shock promoter to produce pGB2-EhClpB. The protein coding sequence of *E. chaffeensis clpB* was amplified from pET28a-EhClpB using AccuTaq LA polymerase MIX (Sigma) with the following PCR primers: CGGCGACGACATATGGATCTCAATCAATTTAC with the NdeI site underlined, and CGGCGACTGCAGTATAACTTATTAATAATTAAA with the PstI site underlined. The *E. coli* σ^32^ promoter sequence was amplified from pGB2-EcClpB [43] using the following primers: CGACCACCCGGGTTCTCGCCTGGTTAGGGC with the XmaI site underlined and CGACGACATATGAACTCCTCCCATAACGGATC with the NdeI site underlined. The PCR products were digested with NdeI, PstI, and XmaI and ligated with the pGB2 plasmid digested with PstI and XmaI to produce pGB2-EhClpB. The *E. coli* strain MC4100ΔclpB [26] was transformed with the empty pGB2, pGB2-EcClpB [43], or pGB2-EhClpB. To test the heat-inducible expression of ClpB, the strains MC4100ΔclpB[pGB2], MC4100ΔclpB[pGB2-EcClpB], and MC4100ΔclpB[pGB2-EhClpB] were grown at 30°C in M9 supplemented with 50 µg/ml spectinomycin to A_578_ = 0.3, then transferred to 45°C for 30 min (heat shock) or maintained at 30°C for 30 min (control) and labeled with ^35^S-methionine (20 µCi/ml, EasyTag, Perkin Elmer) for 10 min. Culture aliquots (0.5 ml) were withdrawn, bacterial cells pelleted and suspended in 50 µl of SDS loading buffer, boiled and analyzed with SDS-PAGE. Autoradiography was performed after the electrotransfer of proteins to nitrocellulose. *E. coli* growth and survival during heat-shock was determined as described before [43].

## Results

### Analysis of the Amino-acid Sequence of EhClpB

AAA+ ATPases contain either one or two ATP-binding AAA+ modules with several conserved sequence motifs, including Walker-A/B and sensor-1/2, and less-conserved additional domains that mediate interactions with substrates or adaptors [24], [25]. The Hsp100 sub-family members contain two AAA+ modules (called D1 and D2) and two less conserved regions: the N-terminal domain (ND) and the middle domain (MD), which is inserted into D1. The sequence alignment of ClpB from *E. chaffeensis* with four other Hsp100 sequences (Fig. S1) revealed that the EhClpB domain organization is similar to those of other Hsp100 proteins and that all characteristic AAA+ motifs are present in the sequence of EhClpB. The sequences of the N-terminal and middle domains are the most divergent among Hsp100 proteins (see Fig. S1). The sequence identity between *E. coli* and *E. chaffeensis* ClpB is 28% within ND, 45% within MD, 75% within D1, and 57% within D2.

### Expression of *E. chaffeensis* clpB During the Infection of Mammalian Cells

**Figure 1:**
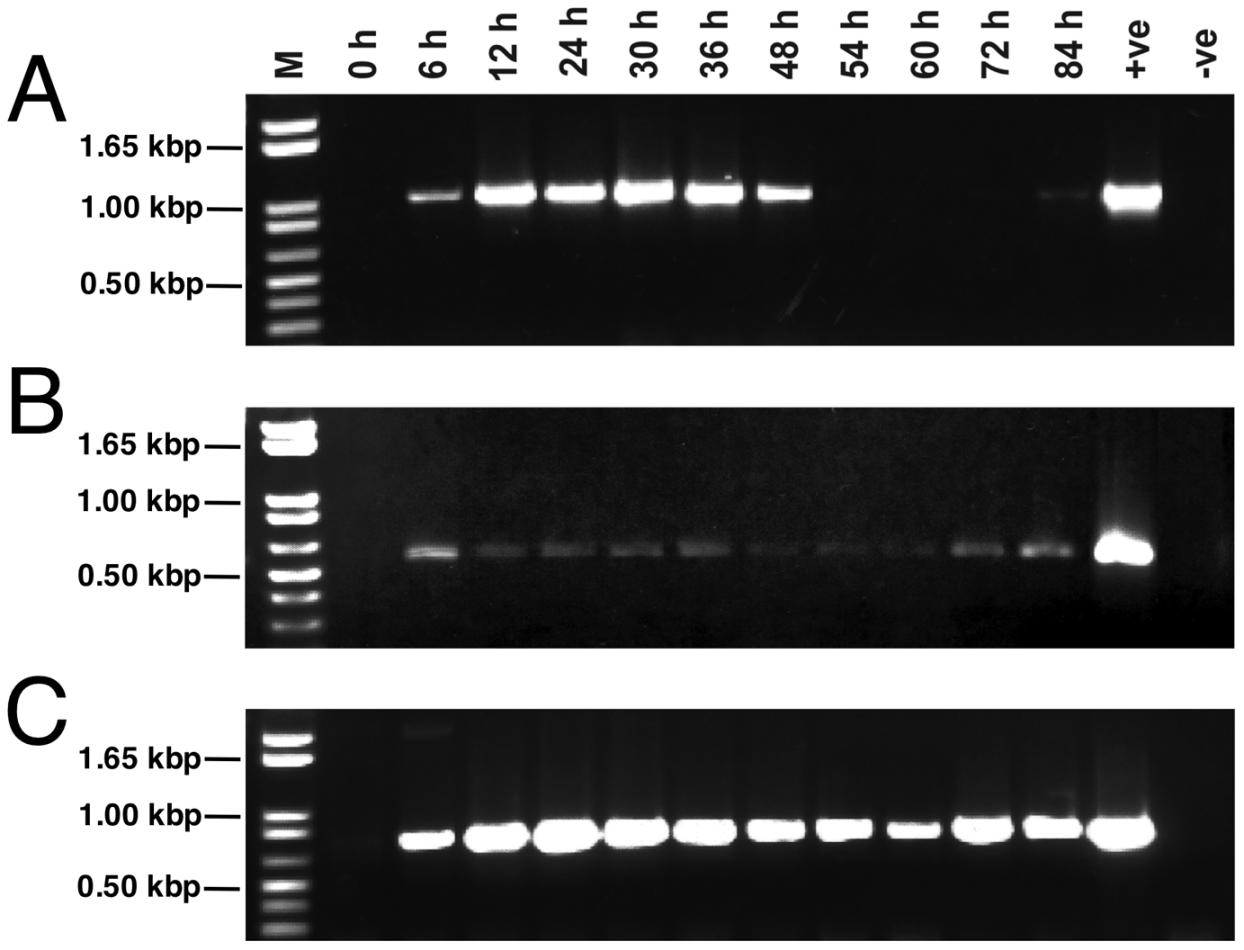
Messenger-RNA levels of the selected molecular chaperones in *E. chaffeensis* during infection of macrophages. *E. chaffeensis* RNA concentration was estimated by real-time RT-PCR targeted to 16 S rRNA and the concentration levels were equalized in all samples assessed. Transcript levels for ClpB (A), DnaK (B), and ClpA (C) were assessed by semi-quantitative RT-PCR using 40 PCR cycles (A, C) or 25 PCR cycles (B). Lanes: M, molecular weight markers; RNA recovered at different time post inoculation of a macrophage culture; +ve, RT-PCR signal from the *E. chaffeensis* genomic DNA (positive control); –ve, RT-PCR in the absence of the DNA template (negative control).

To assess the expression pattern of EhClpB, the *E. chaffeensis* organisms were recovered from the confluent infected macrophage culture and used to infect naïve macrophages. RNA isolated from the culture at different times post infection was examined by semi-quantitative RT-PCR (Fig. 1). The EhClpB transcript level strongly increased during the first 12 h post infection, remained elevated until ∼36 h, and declined thereafter. The EhClpB mRNA signal was almost undetectable after 54 h post infection. In contrast, the transcript levels of ClpA and DnaK showed less variability between 6 and 84 h post infection. The increase in the ClpB mRNA level occurred during the time when the replicating reticulate bodies are typically abundant and the ClpB mRNA declined when reticulate bodies transform to the non-replicating form [44].

### Biochemical Properties of EhClpB

To begin investigating the biological role of ClpB during the infection cycle of *E. chaffeensis*, we produced the recombinant EhClpB in Rosetta BL21(DE3) strain of *E. coli*., which contains tRNAs for the rare Arg codons found in the EhClpB mRNA (assessed with the graphical codon usage analyzer [45]). The recombinant EhClpB in *E. coli* remained soluble and its mobility in the lysate supernatant resolved with SDS-PAGE was consistent with the predicted monomer molecular weight of 95.5 kDa (data not shown). The identity of the purified EhClpB was further confirmed by the mass-spectrometry analysis of tryptic peptides (data not shown).

**Figure 2:**
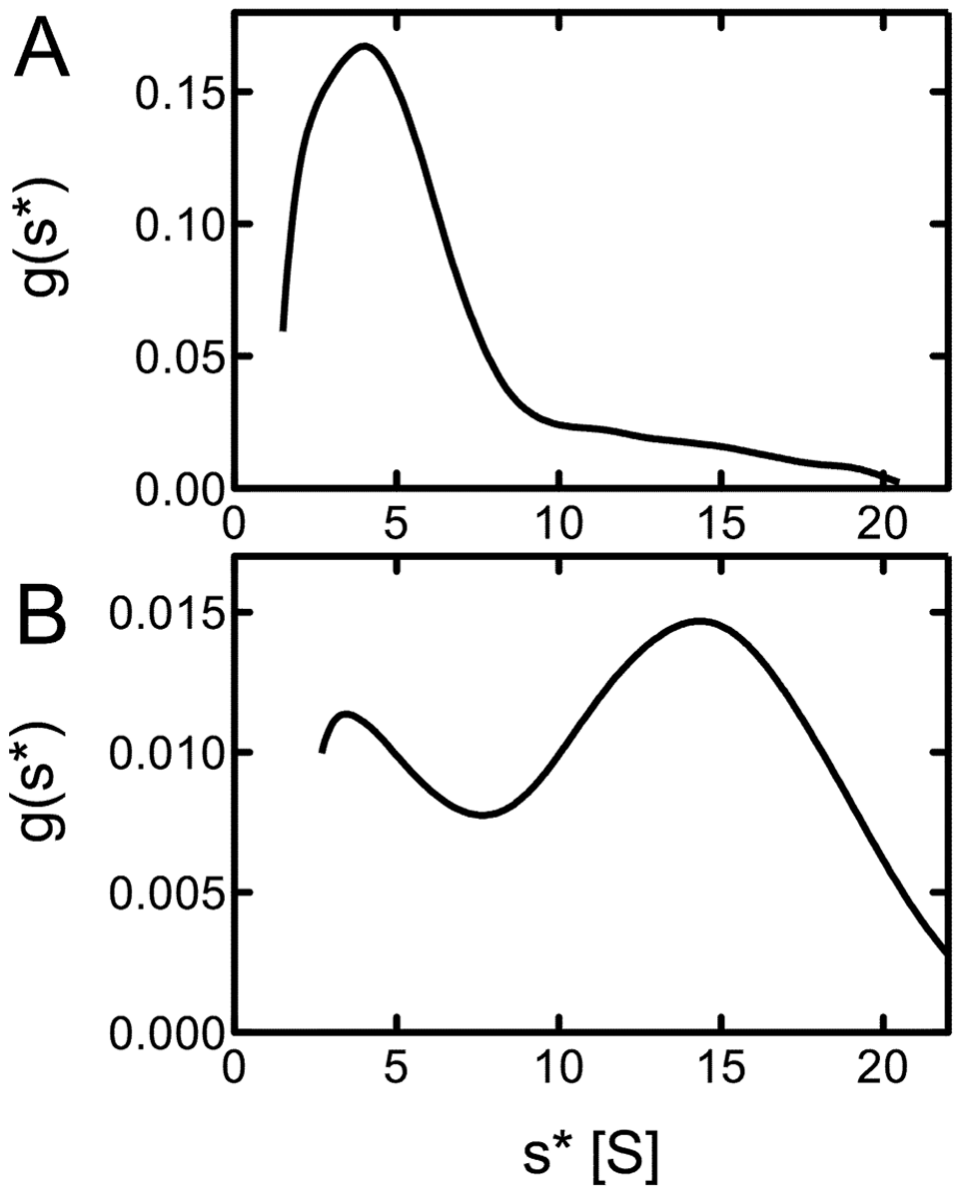
Sedimentation velocity of ClpB from *E. chaffeensis*. Shown are the apparent distributions of the sedimentation coefficient for 0.3 mg/ml EhClpB in the absence of nucleotides (A) and in the presence of 2 mM ATPγS (B).

**Figure 3:**
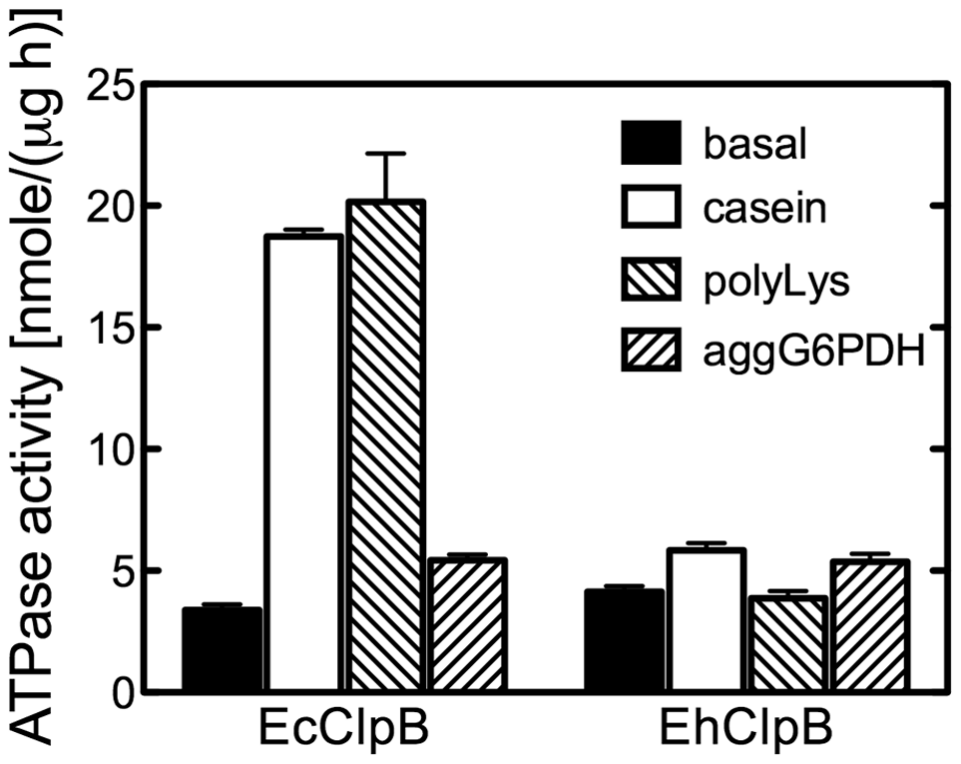
ATPase activity of ClpB from *E. chaffeensis* and *E. coli*. The initial rate of hydrolysis of ATP catalyzed by EhClpB or EcClpB was determined at 37°C in the absence of other proteins (basal activity), with κ-casein, poly-lysine, or aggregated G6PDH. The average values from three separate experiments are shown with the standard deviations.

Since the oligomerization of Hsp100 proteins is linked to their chaperone activity [46], we tested the self-association properties of the recombinant EhClpB using sedimentation velocity. As shown in Fig. 2A, EhClpB in the absence of nucleotides sedimented as a single species with the apparent sedimentation coefficient of ∼4.5 S, which agreed with the previously determined sedimentation coefficient of the monomeric *E. coli* ClpB (EcClpB) [46]. The addition of a non-hydrolyzable ATP analog, ATPγS induced self-association of EhClpB into ∼14.7-S particles (see Fig. 2B), which approximates the sedimentation coefficient of the hexameric EcClpB [47]. We next investigated the ATPase activity of EhClpB. As shown in Fig. 3, the basal ATPase of EhClpB was similar to that of the purified EcClpB. However, while the ATPase of EcClpB was efficiently activated in the presence of casein or poly-lysine, the ATPase of EhClpB did not respond to the putative activators.

### Aggregate-Reactivation Activity of EhClpB

**Figure 4:**
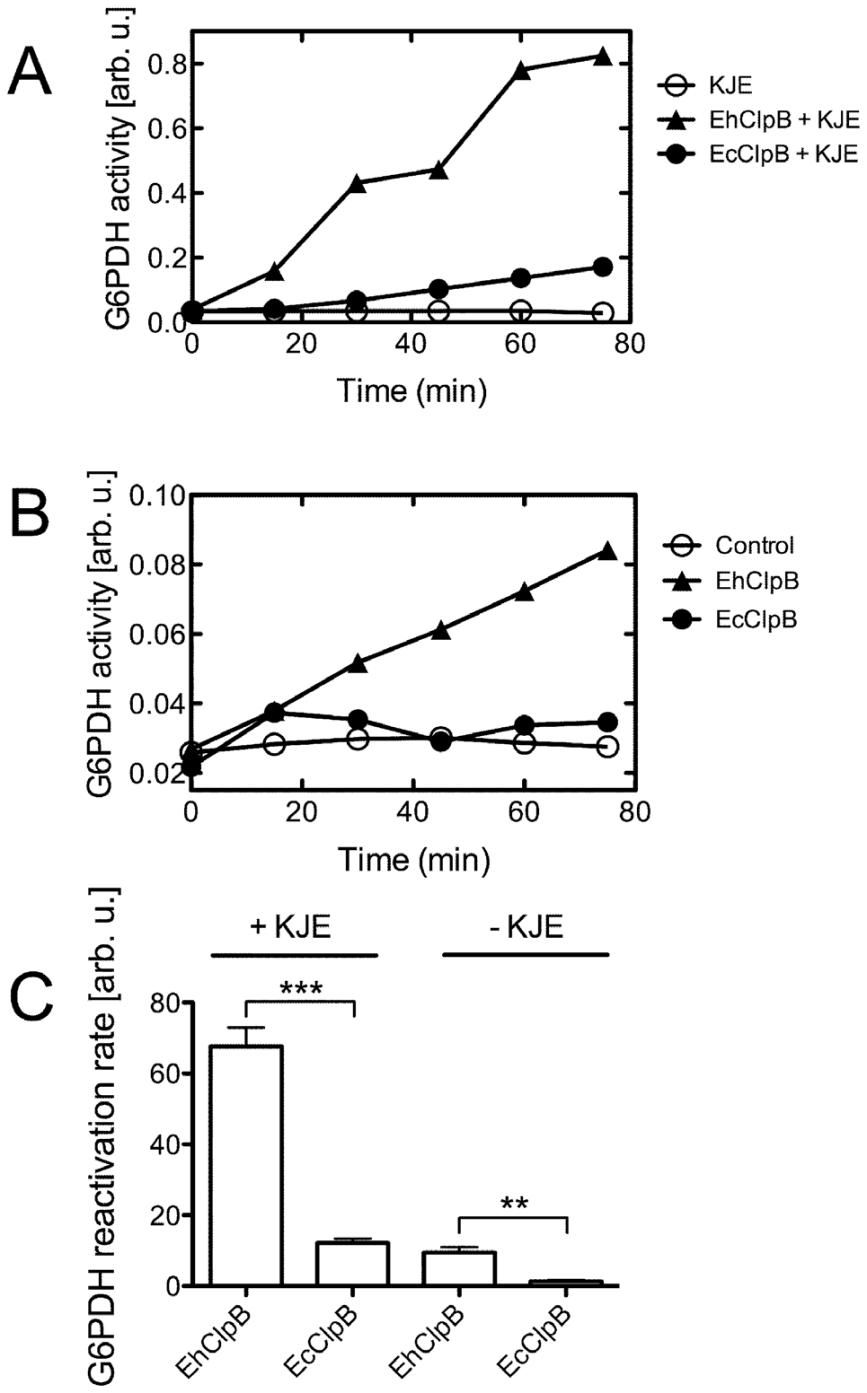
Reactivation of aggregated glucose-6-phosphate dehydrogenase in the presence of ClpB from *E. chaffeensis* and *E. coli*. (A) A representative time-course of the reactivation of aggregated G6PDH in the presence of DnaK/DnaJ/GrpE from *E. coli* without ClpB and with EcClpB or EhClpB. (B) A representative time-course of the reactivation of aggregated G6PDH in the absence of chaperones (control) and in the presence of EcClpB or EhClpB. (C) Initial rates of G6PDH reactivation (from the linear slopes of the data in (A) and (B)). The average values from three independent experiments are shown with the standard deviations. T-test scores: **, p<0.01; ***, p<0.001.

We tested the reactivation of two previously investigated *in vitro* substrates of Hsp100 chaperones: large aggregates produced from chemically denatured glucose-6-phosphate dehydrogenase (G6PDH) [37], [48] and thermally aggregated firefly luciferase [46]. As shown in Fig. 4A, EhClpB reactivated aggregated G6PDH in the presence of the *E. coli* co-chaperones DnaK/DnaJ/GrpE (KJE). The apparent rate of the G6PDH disaggregation was ∼5-fold higher for EhClpB than for EcClpB (see Fig. 4A, C). Unexpectedly, we found that EhClpB reactivated aggregated G6PDH even in the absence of KJE (Fig. 4B) with the reactivation rate close to that found for EcClpB in the presence of the co-chaperones (Fig. 4C).

**Figure 5:**
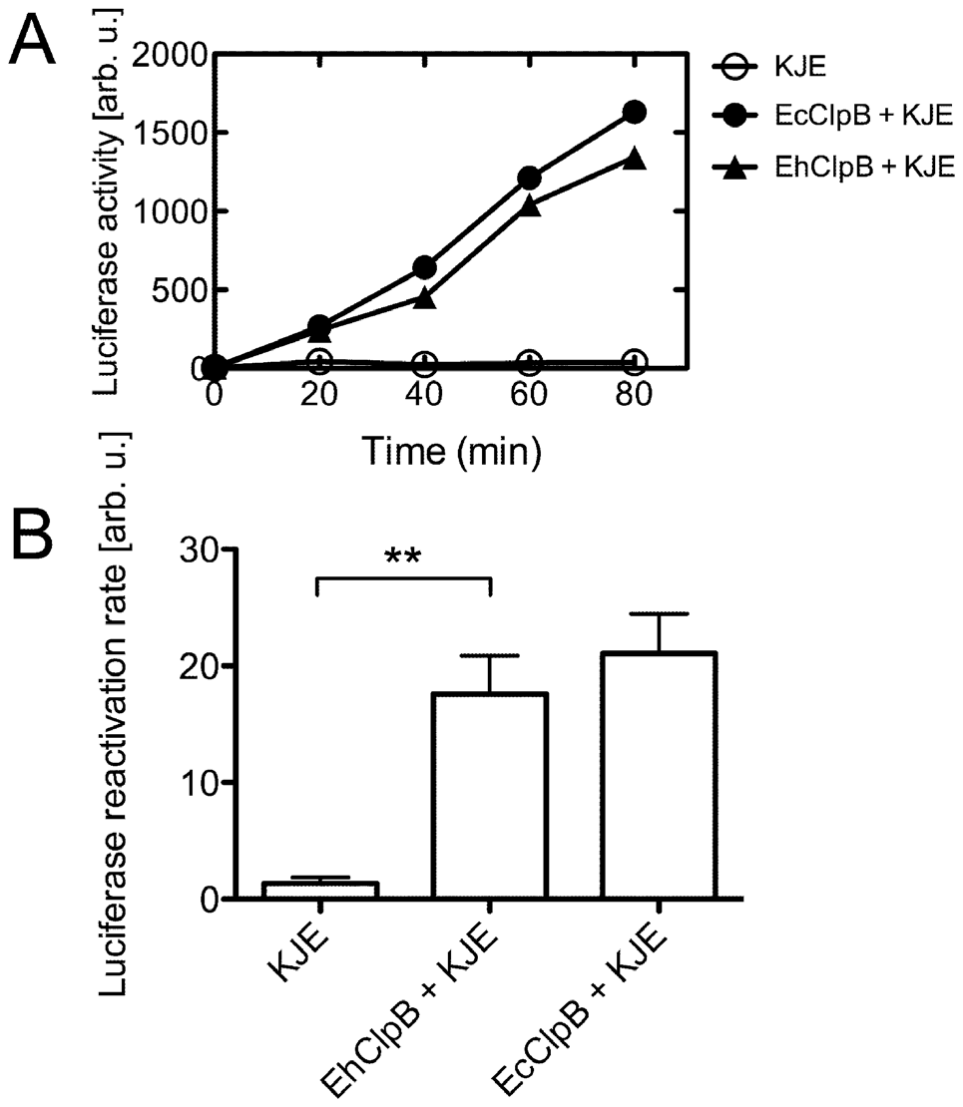
Reactivation of aggregated firefly luciferase in the presence of ClpB from *E. chaffeensis* and *E. coli*. (A) A representative time-course of the reactivation of aggregated luciferase in the presence of DnaK/DnaJ/GrpE from *E. coli* without ClpB and with EcClpB or EhClpB. (B) Initial rates of G6PDH reactivation (from the linear slopes of the data in (A)). The average values from three independent experiments are shown with the standard deviations. T-test scores: **, p<0.01.

EhClpB and EcClpB reactivated aggregated luciferase with similar apparent rates in the presence of KJE (t-test p = 0.5, Fig. 5B). No measurable reactivation of aggregated luciferase was observed in the absence of KJE for either EhClpB or EcClpB (data not shown).

**Figure 6:**
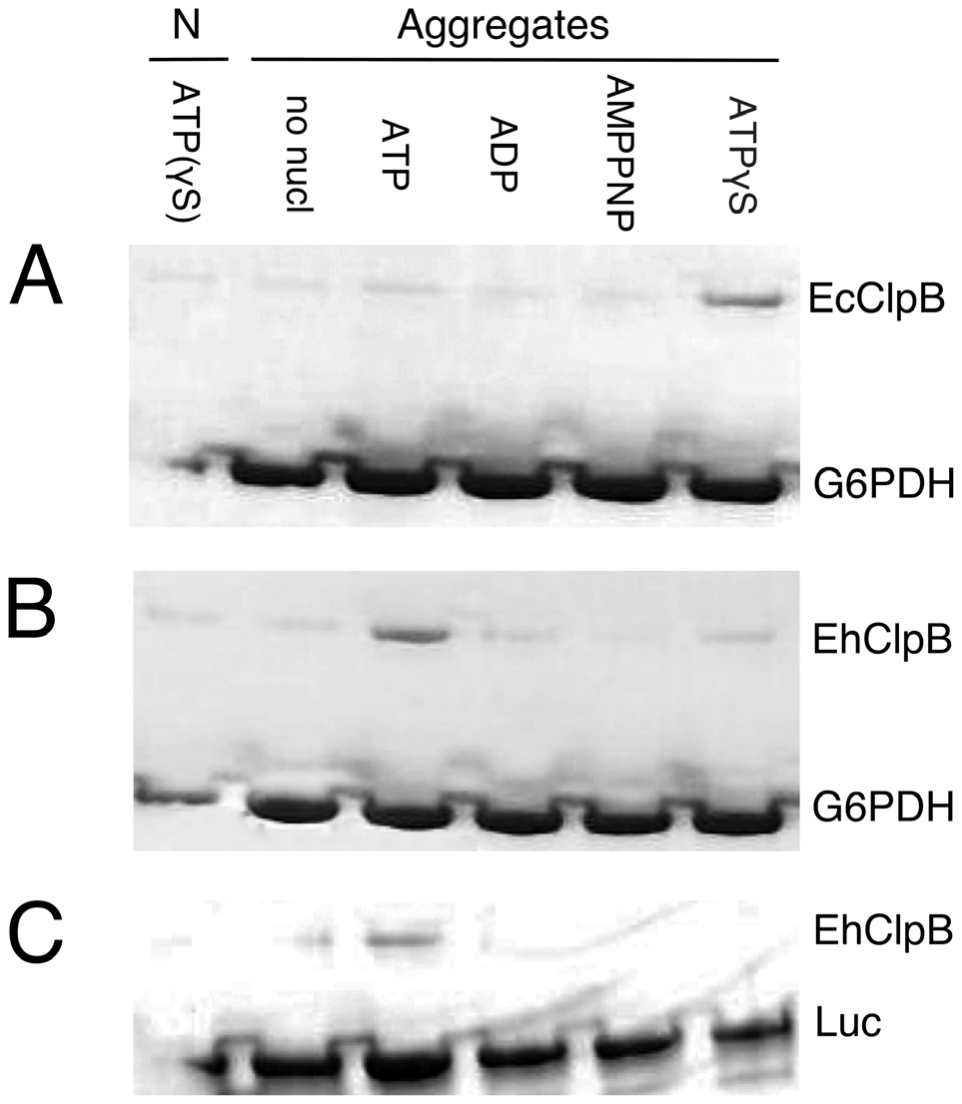
Interactions of ClpB with aggregated substrates. EcClpB (A) or EhClpB (B, C) was incubated with the native (N) or aggregated G6PDH (A, B) or luciferase, Luc (C) in the presence of the indicated nucleotides. The solutions were passed through a 0.1- µm filter and the fractions retained on the filter were analyzed by SDS-PAGE with Coomassie stain. The first lane in (A) shows EcClpB incubated with the native G6PDH and ATPγS, in (B, C) it shows EhClpB incubated with the native G6PDH or luciferase and ATP.

We tested if the apparently higher rate of G6PDH reactivation observed for EhClpB, as compared to EcClpB (see Fig. 4) could be due to a more efficient binding of the *Ehrlichia* chaperone to the aggregated substrate. Previous studies demonstrated that stable interaction of EcClpB with aggregated substrates occurs in the “frozen” ATP-bound state of the chaperone [49], which can be mimicked by supplying a non-hydrolysable ATP analog, ATPγS [50]. We incubated EcClpB and EhClpB with the native or aggregated substrates in the presence of different nucleotides and nucleotide analogs. The aggregates were then separated from soluble proteins using filtration and the aggregate-bound proteins analyzed with SDS-PAGE (Fig. 6). Only background amounts of EcClpB or EhClpB were retained on filters in the absence of the aggregates (first lane in Fig. 6A, B, C). As has been shown before for several aggregated substrates [38], [50], only ATPγS induces significant binding of EcClpB to the aggregates (Fig. 6A). In contrast, we found that EhClpB interacted more efficiently with the aggregated G6PDH and luciferase in the presence of the hydrolysable ATP rather than ATPγS (Fig. 6B, C). We tested if the aggregated G6PDH inhibits the ATP turnover of EhClpB, which could be responsible for the increased stability of the ClpB-substrate complex with ATP. However, as shown in Fig. 3, the rates of ATP hydrolysis supported by EhClpB and EcClpB were similar in the presence of the G6PDH aggregates and were not below the basal rate. Importantly, the amount of aggregate-bound EhClpB in the presence of ATP was similar to that of EcClpB in the presence of ATPγS (Fig. 6A, B), which suggests that the apparent 5-fold difference in the reactivation rate of aggregated G6PDH between EhClpB and EcClpB (Fig. 4) may not be due to a higher affinity of the *Ehrlichia* chaperone towards the aggregated substrate.

### ClpB-Dependent Heat-Shock Survival of E. coli

**Figure 7:**
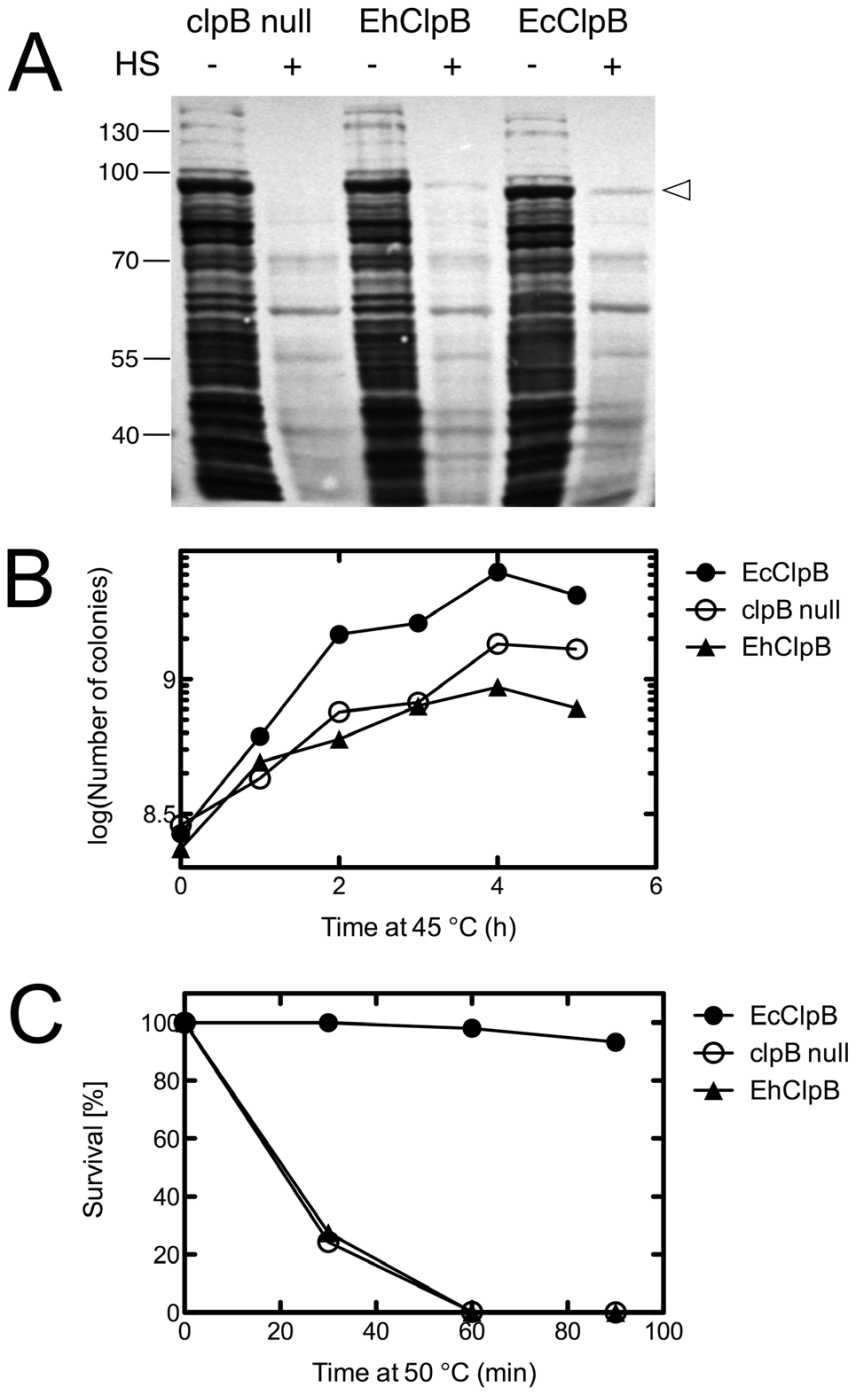
Effect of the production of ClpB from *E. coli* and *E. chaffeensis* on the growth and survival of *E. coli* under heat shock. (A) Autoradiography of the *E. coli* cell lysates obtained from the *clpB*-null strain transformed with pGB2, or with pGB2-EhClpB, or with pGB2-EcClpB, grown at 30°C or 45°C (heat shock conditions, HS). (B) Growth curves of the *clpB*-null strain transformed with pGB2, pGB2-EhClpB, or pGB2-EcClpB at 45°C. (C) Survival of the *clpB*-null strain transformed with pGB2, pGB2-EhClpB, or pGB2-EcClpB, 50°C. The average values from two experiments are shown in (B) and (C).

In *E. coli*, ClpB is dispensable for growth under normal conditions, but becomes essential for survival during heat-shock [26]. We tested if EcClpB can be functionally substituted in *E. coli* with EhClpB. We subcloned the EhClpB DNA sequence into a low-copy pGB2 plasmid and placed it under a control of the native EcClpB heat-shock promoter. Autoradiography of the *E. coli* lysates in Fig. 7A showed that the heat-shock conditions (45°C) induced a prominent expression of a discrete set of proteins, which likely represented the major heat-shock protein families. The Hsp100 signal was absent in the *clpB*-null strain transformed with pGB2, but was present in that strain transformed with pGB2 containing either the EhClpB or EcClpB coding sequence. As reported earlier [26], the lack of a functional ClpB decreased the growth rate of *E. coli* at 45°C (Fig. 7B) and inhibited survival at 50°C (Fig. 7C). Interestingly, the heat-shock-inducible production of EhClpB (Fig. 7A) did not rescue the temperature-sensitive phenotypes in the *clpB*-null *E. coli* (Fig. 7B, C).

## Discussion

In this work, we performed the first biochemical characterization of an Hsp100 chaperone ClpB from *E. chaffeensis*. The amino-acid sequence of EhClpB contains two modules (D1, D2) with several motifs characteristic for the AAA+ superfamily: Walker-A/B and sensor-1/2 (Fig. S1). In the previously studied Hsp100 proteins, the D1 and D2 modules form an ATP-dependent molecular “engine”, which drives the processing of substrates. The sequence of each AAA+ module in EhClpB also contains a “pore-loop” motif (see Fig. S1), which is involved in binding and processing of substrates inside the central pore in the hexameric form of Hsp100 [51], [52]. Moreover, like other Hsp100 proteins, EhClpB contains two less conserved sequence regions: the 145-residue-long N-terminal domain and the middle domain inserted into D1 (see Fig. S1). The N-terminal domain of EhClpB contains a conserved triad Thr7, Asp104, Glu110, which supports the aggregate binding and reactivation in EcClpB [37], [53]. The middle domain of EhClpB contains a conserved Tyr505, which supports the functional cooperation between Hsp100 and Hsp70/40 [54]. The Hsp100-like domain organization and the presence of multiple conserved sequence motifs (see Fig. S1) indicate that EhClpB is a *bona fide* Hsp100, which may perform the function of a molecular chaperone in *E. chaffeensis*.

As shown in Fig. 1, a strong induction of the EhClpB mRNA correlates with the *Ehrlichia* infection of macrophages and the pathogen entry into an intense replicative stage inside the host cells. The EhClpB mRNA subsequently declines to very low levels as the pathogen stops replicating and prepares for its release from host cells. The transcript level of EhClpB during the infection cycle is more variable than those of *E. chaffeensis* ClpA and DnaK (see Fig. 1), which are known regulators of protein homeostasis under stressed, but also non-stressed conditions [55]. The expression pattern of EhClpB mRNA suggests that the function of EhClpB is linked to the pathogen’s adjustment to the intracellular phase of its lifecycle and possibly to its response to host-induced stress.

Purified recombinant EhClpB shows the hallmark biochemical features of Hsp100 proteins: it forms nucleotide-induced hexamers and catalyzes the hydrolysis of ATP (see Figs. 2, 3). EhClpB reactivates inactivated aggregated enzymes: G6PDH and luciferase *in vitro* (see Figs. 4, 5). The aggregate reactivation mediated by EhClpB is more efficient in the presence of the co-chaperones DnaK/DnaJ/GrpE (KJE) from *E. coli*, but, unlike EcClpB, EhClpB does not require the co-chaperones to disaggregate G6PDH (see Fig. 4B, C). Two important conclusions can be drawn from the above observation. First, it has been observed before that there is an apparent “species-specificity” in cooperation between Hsp100 and Hsp70/40 during aggregate reactivation and that such specificity is linked to the sequence of the ClpB middle domain [56], [57]. As shown in Figures 4 and 5, EhClpB does functionally cooperate with KJE from *E. coli*. Thus, the apparent differences in the sequence of the middle domain (see Fig. S1) between EhClpB and EcClpB do not break the functional linkage with KJE. Second, it has been argued that Hsp70/40 are required for the Hsp100-mediated aggregate reactivation because they either perform an obligatory disaggregation step upstream of Hsp100 [58] or target Hsp100 to its aggregated substrates [59]. Our results (see Fig. 4B, C) show that EhClpB displays an intrinsic disaggregase activity, which is independent, at least for some substrates, from that of the co-chaperones.

The Hsp100-mediated aggregate reactivation is linked to the extraction of polypeptides from aggregated particles and their translocation through the central pore in the Hsp100 hexamer [60]. Due to a small diameter of the pore, the extracted polypeptides are released from Hsp100 in unfolded conformation and need to refold in order to manifest a biochemical activity. Efficient refolding of unfolded proteins is often dependent on Hsp70/40 [61]. Thus, the reason why some substrates, like luciferase, are not reactivated by EhClpB without KJE is that they may not refold efficiently without a downstream chaperone assistance. In contrast, unfolded G6PDH does apparently spontaneously refold without chaperone assistance and shows enzymatic activity after its disaggregation by EhClpB (see Fig. 4B, C). It is striking that the apparent rate of G6PDH disaggregation with EhClpB without KJE is similar to that found for EcClpB with KJE (see Fig. 4C).

EcClpB does not form stable complexes with aggregated substrates under conditions of the ATP turnover unless the co-chaperones are also present [62]. Indeed, isolation of the EcClpB-aggregate complexes has been only possible in the presence of the non-hydrolysable ATPγS [50], which induces a stable substrate-binding conformation in ClpB. Unexpectedly, we found that EhClpB binds stably to aggregated substrates in the presence of ATP and does so more efficiently than with ATPγS (see Fig. 6). ATPγS does fit into the nucleotide binding sites of EhClpB since it induces the formation of EhClpB hexamers (see Fig. 2). One should note that not all ATP analogs induce strong binding of substrates to Hsp100, as shown in Fig. 6 by the background levels of aggregate binding to either EcClpB or EhClpB in the presence of AMPPNP. Thus, a subtle allosteric linkage connects the nucleotide binding sites and the aggregate binding site(s) in Hsp100. The allosteric link between the ATPγS-occupied nucleotide sites and the aggregate binding site(s) appears less functional in EhClpB than in EcClpB (see Fig. 6B, C).

The apparent switch in nucleotide preference for stable substrate binding between ATPγS in EcClpB and ATP in EhClpB is perhaps the most unexpected difference between the two chaperones. Previous data suggested that Hsp100 without Hsp70/40 could not maintain contact with the surface of an aggregate while hydrolyzing ATP [62]. Our results demonstrate that EhClpB maintains substrate contact without a support of Hsp70/40 in the presence of ATP (see Fig. 6), which possibly translates into the Hsp70/40-independent reactivation of aggregates by EhClpB (see Fig. 4).

Apparent differences between EhClpB and EcClpB are not limited to the linkage between a nucleotide and substrate binding. EhClpB produced in *E. coli* is biochemically functional (see Figs. 2, 3, 4, 5), cooperates well with the *E. coli* co-chaperones *in vitro* (see Figs. 4, 5) but does not rescue the growth-defect of the *clpB*-null *E. coli* under heat-shock (see Fig. 7). The results in Fig. 7 suggest that the protein clients of EcClpB, which aggregate during heat-shock are not rescued by EhClpB. One can speculate that the substrate specificities of EcClpB and EhClpB are different, possibly because of a different nature of stress experienced by both types of bacteria. Importantly, the EhClpB clients in the replicating *Ehrlichia* may need chaperone assistance during infection of mammalian cells and show distinct recognition motifs from the heat-aggregated proteins in *E. coli*. This hypothesis is consistent with the observation that the ATPase of EhClpB fails to respond to the known pseudo-substrates of EcClpB: casein and poly-lysine (see Fig. 3). Altogether, our study revealed several distinct properties of EhClpB. Considering the unique intraphagosomal environment of a phagocytic host cell (monocyte or macrophage) in which *E. chaffeensis* replicates, EhClpB might have evolved to meet specific demands of the pathogen’s survival under the host-induced stress.

Understanding the role of molecular chaperones in pathogen-host interactions has emerged as a novel direction in studies of the mechanisms of infection. Interfering with the function of chaperones may offer an efficient way of controlling the infectivity and survival of pathogens. Among the known molecular chaperones, the Hsp100 family is particularly promising as a potential target for antibiotics because the Hsp100 proteins are not produced in animal cells. We made the first step towards understanding the biological function and mechanism of the Hsp100 disaggregase ClpB from an important human pathogen *E. chaffeensis*. Further characterization of the EhClpB role in the pathogen’s life cycle and its *in vivo* substrates may create opportunities for developing novel approaches in treating ehrlichiosis and possibly other infectious diseases caused by rickettsial pathogens.

## Supporting Information

Figure S1 **Sequence alignment of the ClpB sequences from** ***Ehrlichia chaffeensis*** **,** ***Escherichia coli*** **,** ***Thermus thermophilus*** **,** ***Saccharomyces cerevisiae*** **, and** ***Arabidopsis thaliana*** **.** Structural domains: N-terminal domain (ND), linker, D1, middle domain (MD), and D2 are indicated below the alignment in bold typeface. The domains’ borders were obtained from the crystallographic data for *T. thermophilus* ClpB [Lee S, Sowa ME, Watanabe YH, Sigler PB, Chiu W, et al. (2003) The structure of ClpB: a molecular chaperone that rescues proteins from an aggregated state. Cell 115∶229–240]. The characteristic motifs of AAA+ ATPases are indicated below the alignment in italics.
(PDF)
