# Supplementary material for: Aggregate-Reactivation Activity of the Molecular Chaperone ClpB from Ehrlichia chaffeensis
Source: PLoS One. 2013 May 7;8(5):e62454. doi: 10.1371/journal.pone.0062454 (PMC3646808; doi:10.1371/journal.pone.0062454)
Supplement: Figure S1 — Sequence alignment of the ClpB sequences from Ehrlichia chaffeensis , Escherichia coli , Thermus thermophilus , Saccharomyces cerevisiae , and Arabidopsis thaliana . Structural domains: N-terminal domain (ND), linker, D1, middle domain (MD), and D2 are indicated below the alignment in bold typeface. The domains’ borders were obtained from the crystallographic data for T. thermophilus ClpB [Lee S, Sowa ME, Watanabe YH, Sigler PB, Chiu W, et al. (2003) The structure of ClpB: a molecular chaperone that rescues proteins from an aggregated state. Cell 115∶229–240]. The characteristic motifs of AAA+ ATPases are indicated below the alignment in italics. (PDF) [file pone.0062454.s001.pdf]

Figure S1

```
ClpB_EhrCh       1 -MDLNQFTDMSKNLIMQAQTTAIASGHQSLIPEHLLKVMLDTKDELIELLLTSCG---CD
ClpB_EColi       1 -MRLDRLTNKFQLALADAQSLALGHDNQFIEPLHLMSALLNQEGGSVSPLLTSAG---IN
ClpB_TheThe      1 -MNLERWTQAAREALAQAQVLAQRMKHQAIDLPHLWAVLLKDERSLAWRLLEKAG---AD
HSP104_SacCer    1 MNDQTQFTERALTILTLAQKLASDHQHPQLQPIHILAAFIETPEDGSVPYLQNLIEKGRY
HSP101_AraTha    1 -MNPEKFTHKTNETIATAHELAVNAGHAQFTPLHLAGALISDPTGIFPQAISSAGG--EN
                   <--                                    ND

ClpB_EhrCh      57 IDKIYSDIKLSLSKLPVVSGSGSGHIHLSKEMAQVLEEAISLAKRNQDTYVTVERLLQAL
ClpB_EColi      57 AGQLRTDINQALNRLPQVEGTG-GDVQPSQDLVRVLNLCDKLAQKRGDNFISSELFVLAA
ClpB_TheThe     57 PKALKELQERELARLPKVEGAEVGQYLTSR-LSGALNRAEGLMEELKDRYVAVDTLVLAL
HSP104_SacCer   61 DYDLFKKVVNRNLVRIPQQQPAPAEITPSYALGKVLQDAAKIQKQQKDSFIAQDHILFAL
HSP101_AraTha   58 AAQSAERVINQALKKLPSQSPPPDDIPASSSLIKVIRRAQAAQKSRGDTHLAVDQLIMGL
                                                      ND

ClpB_EhrCh     117 AVVKDTSVYKILLAHGVTPVKLESLILNMRNGSKADTINAEHKFNALKKYAKDITESAMA
ClpB_EColi     116 LESRGT-LADILKAAGATTANITQAIEQMRGGESVNDQGAEDQRQALKKYTIDLTERAEQ
ClpB_TheThe    116 AEATPG---------LPGLEALKGALKELRGGRTVQTEHAESTYNALEQYGIDLTRLAAE
HSP104_SacCer  121 FNDSSIQQIFKEAQVDIEAIKQQALELRGNTRIDSRGADTNTPLEYLSKYAIDMTEQARQ
HSP101_AraTha  118 LEDSQIRDLLNEVGVATARVKSEVEKLRGKEGKKVESASGDTNFQALKTYGRDLVEQAG-
                                 ND                    --><--  linker  --><--   D1

ClpB_EhrCh     177 GKLDPVIGRDEEIRRTMQVLSRRTKNNPVLIGEPGVGKTAIIEGLAQRIVVGDVPVGLRN
ClpB_EColi     175 GKLDPVIGRDEEIRRTIQVLQRRTKNNPVLIGEPGVGKTAIVEGLAQRIINGEVPEGLKG
ClpB_TheThe    167 GKLDPVIGRDEEIRRVIQILLRRTKNNPVLIGEPGVGKTAIVEGLAQRIVKGDVPEGLKG
HSP104_SacCer  181 GKLDPVIGREEEIRSTIRVLARRIKSNPCLIGEPGIGKTAIIEGVAQRIIDDDVPTILQG
HSP101_AraTha  177 -KLDPVIGRDEEIRRVVRILSRRTKNNPVLIGEPGVGKTAVVEGLAQRIVKGDVPNSLTD
                                                      D1 Walker A

ClpB_EhrCh     237 AKIMALDLGMLVAGTKYRGEFEERLKYVINEIVASNGAVILFIDELHTLVGAGATDGAMD
ClpB_EColi     235 RRVLALDMGALVAGAKYRGEFEERLKGVLNDLAKQEGNVILFIDELHTMVGAGKADGAMD
ClpB_TheThe    227 KRIVSLQMGSLLAGAKYRGEFEERLKAVIQEVVQSQGEVILFIDELHTVVGAGKAEGAVD
HSP104_SacCer  241 AKLFSLDLAALTAGAKYKGDFEERFKGVLKEIEESKTLIVLFIDEIHMLMGNGKDD----
HSP101_AraTha  236 VRLISLDMGALVAGAKYRGEFEERLKSVLKEVEDAEGKVILFIDEIHLVLGAGKTEGSMD
                               Pore loop          D1       Walker B

ClpB_EhrCh     297 ASNLLKPALARGEIHCIGATTLDEYRQHIEKDAALARRFQPVFVSESTVNDTISILRGLK
ClpB_EColi     295 AGNMLKPALARGELHCVGATTLDEYRQYIEKDAALERRFQKVFVAEPSVEDTIAILRGLK
ClpB_TheThe    287 AGNMLKPALARGELRLIGATTLDEYR-EIEKDPALERRFQPVYVDEPTVEETISILRGLK
HSP104_SacCer  297 AANILKPALSRGQLKVIGATTNNEYRSIVEKDGAFERRFQKIEVAEPSVRQTVAILRGLQ
HSP101_AraTha  296 AANLFKPMLARGQLRCIGATTLEEYRKYVEKDAAFERRFQQVYVAEPSVPDTISILRGLK
                                   Sensor 1       D1

ClpB_EhrCh     357 EKYEVHHGIRIMDSAIIAASTLSNRYITDRFLPDKAIDLIDEAASRVRIEIDSKPEVIDE
ClpB_EColi     355 ERYELHHHVQITDPAIVAAATLSHRYIADRQLPDKAIDLIDEAASSIRMQIDSKPEELDR
ClpB_TheThe    346 EKYEVHHGVRISDSAIIAAATLSHRYITERRLPDKAIDLIDEAAARLRMALESAPEEIDA
HSP104_SacCer  357 PKYEIHHGVRILDSALVTAAQLAKRYLPYRRLPDSALDLVDISCAGVAVARDSKPEELDS
HSP101_AraTha  356 EKYEGHHGVRIQDRALINAAQLSARYITGRHLPDKAIDLVDEACANVRVQLDSQPEEIDN
                                                      D1                      --><--

ClpB_EhrCh     417 LDRKIIQLKIEAGVLEKEN--TESSKQRLAQLSEELNKLSIQATELNSKWQAEKMKILKM
ClpB_EColi     415 LDRRIIQLKLEQQALMKES--DEASKKRLDMLNEELSDKERQYSELEEEWKAEKASLSGT
ClpB_TheThe    406 LERKKLQLEIEREALKKEK--DPDSQERLKAIEAEIAKLTEEIAKLRAEWEREREILRKL
HSP104_SacCer  417 KERQLQLIQVEIKALERDEDADSTTKDRLKLARQKEASLQEELEPLRQRYNEEKHGHEEL
HSP101_AraTha  416 LERKRMQLEIELHALEREK--DKASKARLIEVRKELDDLRDKLQPLTMKYRKEKERIDEI
                                                      MD

ClpB_EhrCh     475 QECVEKLDNARNDLEKAQRSGNLAKAGELMYGIIPELEKELKKCEKPS---------SNM
ClpB_EColi     473 QTIKAELEQAKIAIEQARRVGDLARMSELQYGKIPELEKQLEAATQLEGKT------MRL
ClpB_TheThe    464 REAQHRLDEVRREIELAERQYDLNRAAELRYGELPKLEAEVEALSEKLRG-------ARF
HSP104_SacCer  477 TQAKKKLDELENKALDAERRYDTATAADLRYFAIPDIKKQIEKLEDQVAEEERRAGANSM
HSP101_AraTha  474 RRLKQKREELMFSLQEAERRYDLARAADLRYGAIQEVESAIAQLEGTSSEE------NVM
                                                      MD                          -->
```

Figure S1 (continued)

```
ClpB_EhrCh     526 LKREVTESDIASIVSRWTGIPIENMMSSEKEKLLRMEEEIGKTVIGQESAIKAVSDAVRR
ClpB_EColi     527 LRNKVTDAEIAEVIARWTGIPVSRMMESEREKLLRMEQELHHRVIGQNEAVDAVSNAIRR
ClpB_TheThe    517 VRLEVTEEDIAEIVSRWTGIPVSKLLEGEREKLLRLEEELHKRVVGQDEAIRAVADAIRR
HSP104_SacCer  537 IQNVVDSDTISETAARLTGIPVKKLSESENEKLIHMERDLSSEVVGQMDAIKAVSNAVRL
HSP101_AraTha  528 LTENVGPEHIAEVVSRWTGIPVTRLGQNEKERLIGLADRLHKRVVGQNQAVNAVSEAILR
                   <--      D1     --><--                   D2

ClpB_EhrCh     586 SRAGVQDANKPLGSFLFLGPTGVGKTELVKTLAEFLFCDKSALLRFDMSEFMEKHAVSRL
ClpB_EColi     587 SRAGLADPNRPIGSFLFLGPTGVGKTELCKALANFMFDSDEAMVRIDMSEFMEKHSVSRL
ClpB_TheThe    577 ARAGLKDPNRPIGSFLFLGPTGVGKTELAKTLAATLFDTEEAMIRIDMTEYMEKHAVSRL
HSP104_SacCer  597 SRSGLANPRQPAS-FLFLGLSGSGKTELAKKVAGFLFNDEDMMIRVDCSELSEKYAVSKL
HSP101_AraTha  588 SRAGLGRPQQPTGSFLFLGPTGVGKTELAKALAEQLFDDENLLVRIDMSEYMEQHSVSRL
                                     Walker A  D2

ClpB_EhrCh     646 IGAPPGYVGYDQGGMLTESVRRRPYQVILFDEIEKAHGDIFNILLQVLDEGRLTDNHGKL
ClpB_EColi     647 VGAPPGYVGYEEGGYLTEAVRRRPYSVILLDEVEKAHPDVFNILLQVLDDGRLTDGQGRT
ClpB_TheThe    637 IGAPPGYVGYEEGGQLTEAVRRRPYSVILFDEIEKAHPDVFNILLQILDDGRLTDSHGRT
HSP104_SacCer  656 LGTTAGYVGYDEGGFLTNQLQYKPYSVLLFDEVEKAHPDVLTVMLQMLDDGRITSGQGKT
HSP101_AraTha  648 IGAPPGYVGHEEGGQLTEAVRRRPYCVILFDEVEKAHVAVFNTLLQVLDDGRLTDGQGRT
                      Pore loop                Walker B D2

ClpB_EhrCh     706 VDFRNTILVLTSNLGQEILINNKED-VDGESVKKSITSVLQHHFRPEFLNRLDEIIVFHR
ClpB_EColi     707 VDFRNTVVIMTSNLGSDLIQERFGE-LDYAHMKELVLGVVSHNFRPEFINRIDEVVVFHP
ClpB_TheThe    697 VDFRNTVIILTSNLGSPLILEGLQKGWPYERIRDEVFKVLQQHFRPEFLNRLDEIVVFRP
HSP104_SacCer  716 IDCSNCIVIMTSNLGAEFINSQQGS-KIQESTKNLVMGAVRQHFRPEFLNRISSIVIFNK
HSP101_AraTha  708 VDFRNSVIIMTSNLGAEHLLAGLTGKVTMEVARDCVMREVRKHFRPELLNRLDEIVVFDP
                               Sensor 1        D2

ClpB_EhrCh     765 LTKEHIEKIIDVQFSLLQKIVAQ--KELEISLSSEAKSWLMNNGYDSLYGARPLKRLIQQ
ClpB_EColi     766 LGEQHIASIAQIQLKRLYKRLEE--RGYEIHISDEALKLLSENGYDPVYGARPLKRAIQQ
ClpB_TheThe    757 LTKEQIRQIVEIQLSYLRARLAE--KRISLELTEAAKDFLAERGYDPVFGARPLRRVIQR
HSP104_SacCer  775 LSRKAIHKIVDIRLKEIEERFEQNDKHYKLNLTQEAKDFLAKYGYSDDMGARPLNRLIQN
HSP101_AraTha  768 LSHDQLRKVARLQMKDVAVRLAE--RGVALAVTDAALDYILAESYDPVYGARPIRRWMEK
                                               D2              Sensor 2

ClpB_EhrCh     823 KIQNSLAKLIILANQVSKGDKLEVVLLNDDLIINKL------------------------
ClpB_EColi     824 QIENPLAQQILSGELVPGKVIRLEVNEDRIVAVQ--------------------------
ClpB_TheThe    815 ELETPLAQKILAGEVKEGDRVQVDVGPAGLVFAVPARVEA--------------------
HSP104_SacCer  835 EILNKLALRILKNEIKDKETVNVVLKKGKSRDENVPEEAEECLEVLPNHEATIGADTLGD
HSP101_AraTha  826 KVVTELSKMVVREEIDENSTVYIDAGAGDLVYRVESGGLVDASTGKKSDVLIHIANGPKR
                                               D2

ClpB_EhrCh         --------------------------
ClpB_EColi         --------------------------
ClpB_TheThe        --------------------------
HSP104_SacCer  895 DDNEDSMEIDDDLD------------
HSP101_AraTha  886 SDAAQAVKKMRIEEIEDDDNEEMIED
                        D2                -->
```
